# RoadDiffBox: Automatic Road Distress Diagnosis through Controlled Image Generation and Semi-Supervised Learning

**DOI:** 10.34133/research.0833

**Published:** 2025-08-25

**Authors:** Yuanyuan Hu, Ning Chen, Hancheng Zhang, Yue Hou, Pengfei Liu

**Affiliations:** ^1^ Institute of Highway Engineering, RWTH Aachen University, Aachen, Germany.; ^2^ Beijing Key Laboratory of Traffic Engineering, Beijing University of Technology, Beijing, China.; ^3^ Department of Civil Engineering, Faculty of Science and Engineering, Swansea University, Swansea, UK.

## Abstract

During the designed service life, road infrastructures will bear repeated loading conditions from vehicle weights and environmental conditions, resulting in the inevitable occurrence of road distresses including cracks, potholes, etc. The traditional inspection methods by transportation engineers are normally costly and labor-intensive. In recent years, artificial intelligence (AI)-based road distress detection methods have been widely used as convenient and automated approaches, while the AI-based methods heavily depend on a large amount of high-quality images, limiting the real engineering applications. To address the issues, this study introduces RoadDiffBox, a novel framework employing controlled image generation and semi-supervised learning. The framework addresses dataset imbalances through class control and accelerates image generation by utilizing the denoising diffusion implicit model’s reverse process sampling method, while employing knowledge distillation techniques optimized for resource-constrained mobile devices. It generates diverse and high-quality road distress images with automatic bounding box annotations, substantially reducing manual labeling requirements. Test results show that RoadDiffBox demonstrates strong generalizability across geographic regions (Germany, China, and India) and shows cross-domain potential in medical imaging applications. Performance evaluations demonstrate RoadDiffBox’s effectiveness, with classification models achieving an F1-score of 0.95 and detection models reaching a mean average precision (mAP@50) of 0.95 and an F1-score of 0.91 in controlled settings, while maintaining robust performance (an F1-score of 0.86 and a mAP@50 of 0.91) during on-site testing in real-world conditions. On server-class hardware, the model achieves generation times as low as 0.18 s per image. It is discovered that RoadDiffBox can serve as a scalable and efficient solution for real-time road maintenance with limited datasets.

## Introduction

The structural integrity, service quality, and functional performance of roadway infrastructure are critical to ensuring public transportation safety [1]. Under long-term servicing in severe environmental conditions, there will occur inevitable distresses in the road infrastructures, including various kinds of pavement damages such as cracks and potholes, posing serious risks to vehicle operation and public safety [2]. If these problems are not addressed appropriately, the deteriorations can accelerate the overall degradation of infrastructure, leading to more extensive repair costs and higher structural failure risks [3,4]. Accurate and efficient road distress detection is crucial for modern pavement management systems, enabling precise condition assessments and informed maintenance planning decisions. Timely maintenance to detect road distresses before they develop into structural failures by the transportation engineers are, therefore, essential to mitigating these impacts and extending the service life of road networks. As road infrastructure systems worldwide reach or surpass their designed service life, countries across the globe are increasingly prioritizing large-scale road maintenance initiatives [5]. However, traditional inspection methods, typically reliant on labor-intensive and manual assessments by transportation engineers, are costly and time-consuming [6], indicating an urgent need for innovative solutions to reduce inspection and maintenance costs while improving efficiency.

Recently, there have been many engineering demonstrations and applications for real road distresses detection using artificial intelligence (AI) worldwide. However, there exist a major problem for AI-based road distress detection tasks; i.e., training a reliable detection model normally requires hundreds of thousands of high-quality images [7,8], while the traditional labor-intensive and costly nature of manual annotation for crack detection by experienced transportation engineers result in a substantial challenge in obtaining large and high-quality labeled datasets [9,10] of road distresses. Various methodological approaches have been proposed to address this fundamental challenge, including data augmentation techniques across different sensor modalities. These encompass traditional image-based augmentation methods [11–14], acoustic signal processing techniques for structural health monitoring [15,16], and vibration-based data enhancement strategies [17,18]. However, in the context of road distress detection, image-based augmentation remains the predominant approach due to the visual nature of pavement damage assessment. Neverheless, these traditional image augmentation techniques, while enhancing model robustness through geometric transformations and noise injection, cannot generate examples beyond the existing data distribution, limiting their ability to address substantial data gaps or imbalances in road distress categories. Another approach is domain adaptation in transfer learning [19–21], which utilizes knowledge from other domains to enhance the accuracy of road crack detection, reduce training time and computational resources, and improve model generalization by incorporating knowledge from diverse fields. Despite its success in various areas, this method faces challenges in detecting road distress, as cracks and potholes differ markedly from typical objects in standard datasets. This fundamental domain gap limits effective knowledge transfer and necessitates alternative approaches that can better use available data resources. Semi-supervised learning methodologies [22–25] present a cost-effective approach to road distress detection by simultaneously utilizing labeled and unlabeled data. This approach is particularly valuable for road infrastructure monitoring applications, where expert-labeled data are both scarce and expensive to obtain. Through techniques such as pseudo-labeling, consistency regularization, and self-training, semi-supervised learning facilitates model training with minimal manual annotation requirements. Despite these advantages, the method faces substantial challenges when applied to road distress detection. The inherent visual characteristics of pavement damage—including irregular morphology, elongated patterns, and low contrast against the background—substantially complicate the pseudo-labeling process. Consequently, generated pseudo-labels often contain errors that introduce noise into the training pipeline. These inaccuracies can propagate through the model training process, ultimately degrading detection performance and limiting the practical utility of semi-supervised approaches for road distress applications. Generative models like variational autoencoders (VAEs) [26,27] and generative adversarial networks (GANs) [28] create synthetic crack images. VAEs often produce blurry images, limiting their utility for fine-grained crack detection, while GANs, despite producing realistic images, suffer from issues like mode collapse and artifacts [29]. Additionally, neither model can readily generate accompanying ground-truth annotations, such as bounding boxes, which are essential for training robust object detection models.

To address the challenge of generating high-quality datasets, the concept of AI-generated content (AIGC) [30] has emerged, bringing potential to fields like image generation and enhancement [31]. AIGC employs advanced AI techniques to automatically create diverse and high-quality content. Among these techniques, diffusion models have gained attention as a state-of-the-art—referring to the highest level of achievement in the field at the current time—approach in image generation, demonstrating outstanding performance in producing high-resolution and complex images for applications such as medical imaging, autonomous driving, and creative arts [32,33]. Akrout et al. [34] demonstrated that synthetic images from diffusion models enhance skin classifier accuracy, with hybrid synthetic–real data training outperforming single-source approaches. Universal transformer diffusion modeling for vulnerable pedestrian trajectory prediction (UTD-PTP) [35] successfully predicts vulnerable pedestrian trajectories in complex environments, while Diffusion-ES [36] optimizes black-box objectives while preserving trajectory naturalism in autonomous driving applications. In road engineering, diffusion models show promise for generating synthetic images of road conditions to enhance distress detection datasets. Cano-Ortiz et al. [37] proposed a generative diffusion model for data augmentation that produces synthetic images of rare defects, investigating methods to enhance image quality and reduce production time. Leveraging a diffusion model—crack diffusion model (CDM)—generates synthetic cracks with controlled morphology and positioning, enhancing detection and segmentation in complex environments [38]. Despite the promising potential of AIGC for road distress detection, several substantial limitations hinder its widespread application in real-world scenarios. Most current diffusion models lack effective class control, making it difficult to guide the generation process toward specific types of road distresses, leading to dataset imbalances with certain crack types overrepresented while others are underrepresented. This creates a long-tail problem where rare distress categories appear infrequently in the dataset, challenging model training and generalization. Additionally, while diffusion models can produce synthetic images, these images typically require manual annotation for object detection tasks that need bounding boxes or segmentation masks, which reintroduces high annotation costs and negates a primary advantage of AIGC approaches. Furthermore, conventional diffusion models suffer from slow generation speed during computations, with single image production taking minutes, making these models impractical for large-scale applications. This computational burden, coupled with high resource demands, makes diffusion models difficult to deploy in real-time scenarios or on servers with limited computational capabilities, particularly for urgent road distress detection tasks by transportation engineers.

Evaluating the progression of road distress detection approaches from traditional techniques to advanced generative methods reveals substantial advancements alongside persistent challenges in each methodology. Table provides a comprehensive summary of related works on image-based data augmentation for road distress detection, highlighting their relative strengths and limitations.

**Table.** Summary of related works in image-based data augmentation for road distress detection. Icons by Larea, fatimahazzahra, and BEARicons from The Noun Project (CC BY 3.0).

Category	Publication	Method	Limitation
Traditional data augmentation techniques 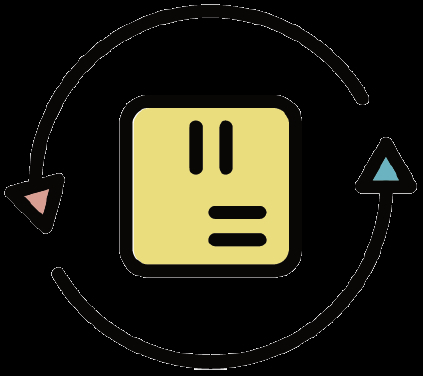	Chen et al. [11], Xu et al. [12]	Traditional augmentation was done through rotation and flipping	Remaining confined to the existing data distribution limits the model generalization. This makes it challenging to capture region-specific variations in distress patterns
	Wang et al. [13], Ouma and Hahn [14]	Color adjustment and geometric transformations were applied for data augmentation to enhance dataset quality and diversity	
Domain adaption in transfer learning 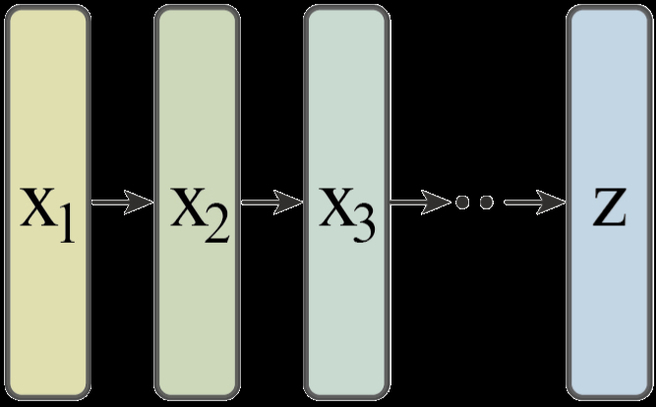	Li et al. [19], Wu et al. [20]	Transferring existing image data to new scene styles, followed by domain adaptation to apply extracted features to detection in the new scenario	Facing challenges in road distress detection, this method struggles with domain gaps. Substantial differences between cracks, potholes, and typical objects in standard datasets limit effective knowledge transfer.
	Zhang et al. [21]	Leveraging stagewise domain adaption RoadDA aligns features and refines intradomain consistency	
Semi-supervised learning 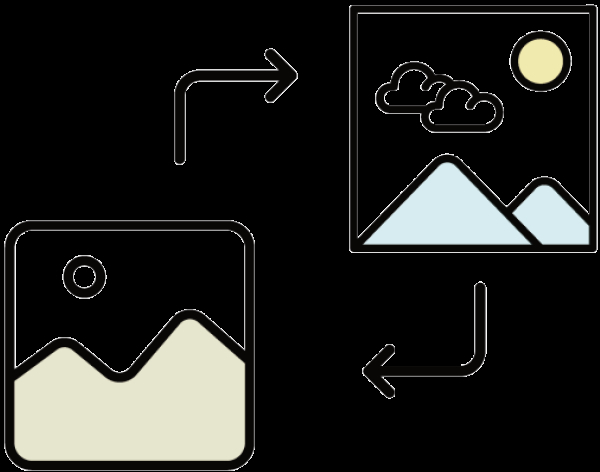	Shamsabadi et al. [22], He et al. [23]	The framework combines consistency regularization and certainty-based self-training to enhance accuracy with limited labeled data.	Ensuring pseudo-label reliability in road distress detection is challenging, as irregular, low-contrast cracks and potholes introduce noise, leading to error propagation and performance degradation.
	Arce-Sáenz et al. [24], Tao [25]	Employing semi-supervised training, the method uses pseudo-labels for model retraining to enhance performance.	
Variational autoencoders (VAEs) 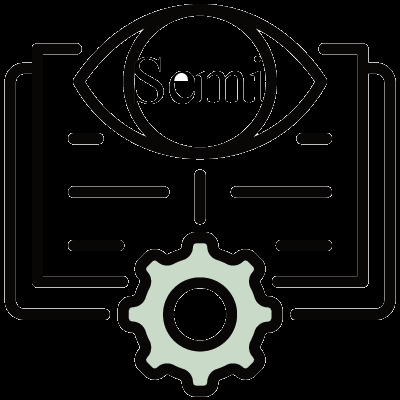	Hu et al. [26]	Utilizing 4 VAE models, DG-MMF augments bearing fault data by generating multiscale features for improved diagnosis.	Generating inherently blurry images, VAEs struggle with fine-grained crack detected. This reduces their effectiveness in capturing detailed distress patterns.
	Faber et al. [27]	Introducing VLAD, a VAE-based lifelong anomaly detection method with hierarchical memory and adaptive model updates.	
Generative adversarial networks (GANs) 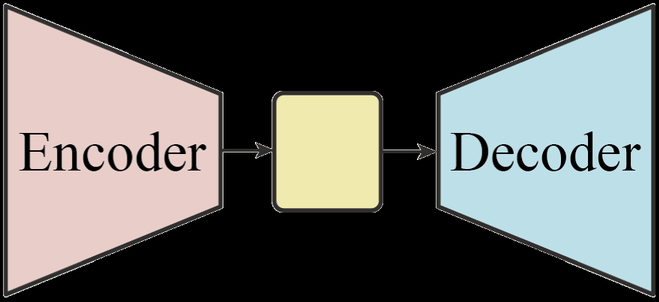	Zhang et al. [28]	Proposing a commonsense-driven GAN to generate photo-realistic images based on entity-related knowledge.	While generating realistic images, GANs struggle with mode collapse and artifacts. They cannot produce essential ground-truth annotations like bounding boxes for object detection.
	Yang et al. [59]	AG-GAN generates virtual pavement images with segregation failure, enabling enhanced analysis and evaluation.	
Diffusion models 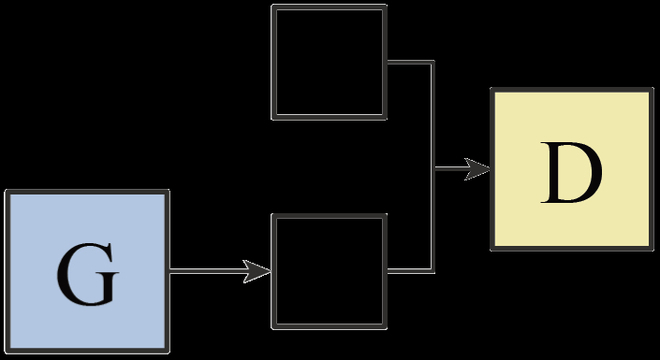	Cano-Ortiz et al. [37]	A generative diffusion model enhances data augmentation by synthesizing rare defect images with improved quality and efficiency.	Slow generation speed, limited class control, and the need for manual annotation. These restrict the practical application of diffusion models in real-world engineering.
	Zhang et al. [38]	CDM enhances crack synthesis with precise control over position and morphology improving detection and segmentation.	

In this paper, RoadDiffBox is presented as a lightweight diffusion model specifically designed to address the challenges of road distress generation and detection. The name reflects its application to road infrastructure (“Road”), diffusion model architecture (“Diff”), and automated bounding box annotation capability (“Box”). The model provides several key contributions:

1. RoadDiffBox offers controlled generation of various crack types, effectively creating balanced datasets and mitigating the long-tail problem where certain distress categories are underrepresented, thereby resolving classification challenges in road distress detection by ensuring adequate representation of all damage categories in the training data.
2. The model automatically produces corresponding bounding boxes during image generation, eliminating manual annotation requirements and enabling semi-supervised learning techniques with minimal labeling costs, effectively addressing the object detection challenges in road distress analysis by providing precise localization data for training robust detection models without human intervention.
3. The framework employs denoising diffusion implicit model (DDIM) sampling to accelerate image generation and applies knowledge distillation techniques for model compression, allowing efficient deployment on devices with varying computational capacities.

## Results

**Fig. 1.**
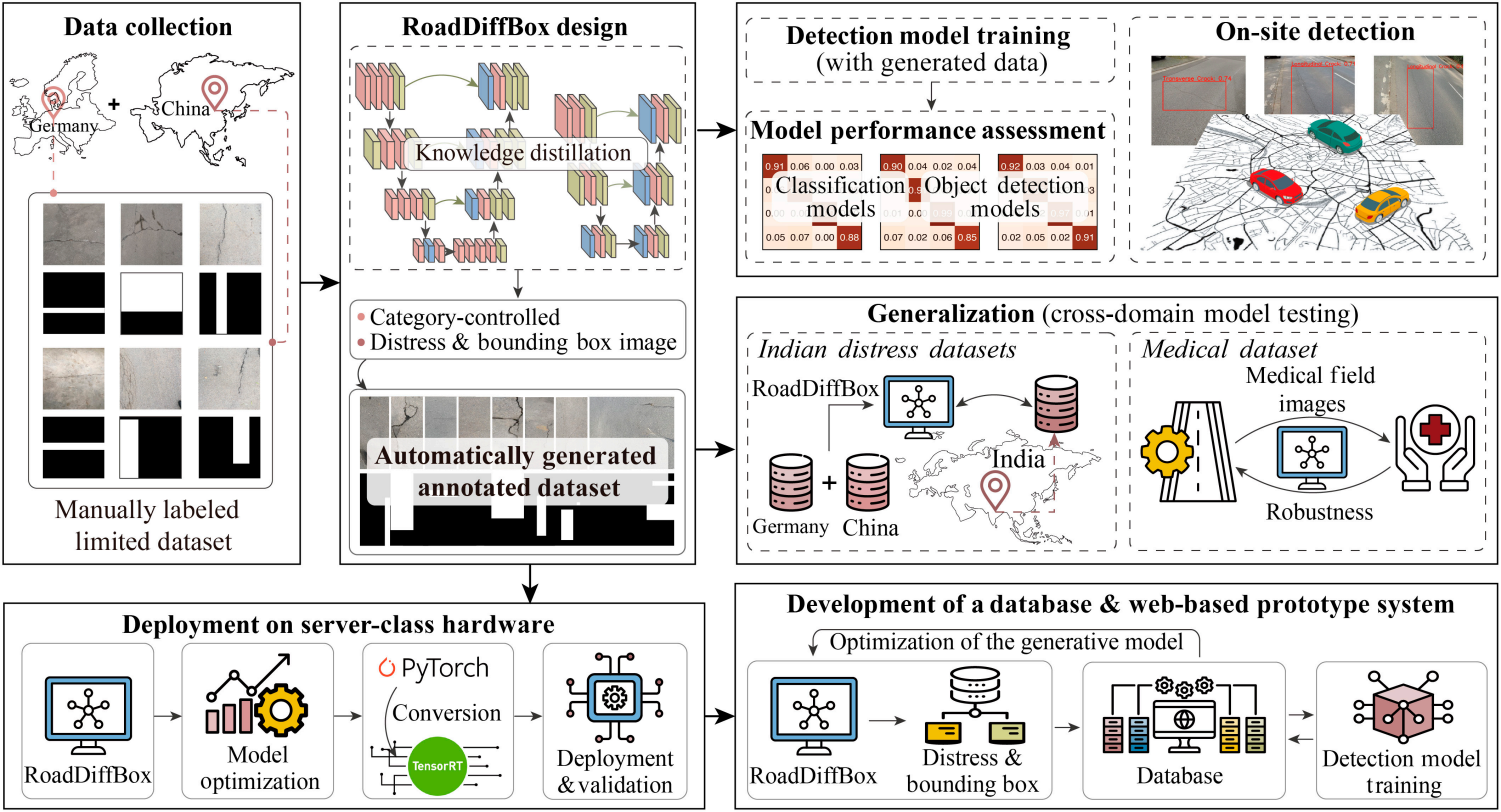
Overview of the RoadDiffBox workflow for road distress image generation and detection. Icons by Rahe, Naba A’la Lail, Naba A’la Lail, nangicon, Putri Creative, and okta from The Noun Project (CC BY 3.0). Map tiles adapted from Stamen Design under CC BY 3.0. Data © OpenStreetMap contributors (ODbL).

This study’s workflow is divided into 6 main stages, as shown in Fig. 1, which illustrates the complete pipeline from limited data collection to real-world deployment:

1. Data Collection: Road distress images were gathered from Germany (Aachen) and China (Beijing) to capture common crack development patterns caused by different environmental and structural factors. This initial dataset requires costly manual annotation by transportation engineers for accurate labeling. The dataset is categorized into 4 types of cracks: longitudinal cracks, alligator cracks, repaired pavement, and transverse cracks. This manually labeled dataset provides essential ground truth and serves as the foundation for subsequent data augmentation through the RoadDiffBox framework.
2. Design of the RoadDiffBox Framework: RoadDiffBox serves as the core generative model that addresses the data scarcity challenge. The model is trained on the limited manually labeled dataset to learn road distress patterns and characteristics. Once trained, RoadDiffBox can generate diverse road distress images by simply specifying the desired crack type through class control parameters. The framework employs knowledge distillation to optimize the diffusion process, enabling efficient production of synthetic images with automatically generated bounding box annotations, thereby substantially expanding the available training data for detection model development.

3. Detection Model Training and Evaluation: The automatically generated annotated dataset from RoadDiffBox is utilized to train various detection and classification models, demonstrating how synthetic data can effectively substitute for large-scale manual annotation. Comparative evaluations are conducted between models trained on the augmented dataset versus those trained on the original limited dataset, revealing substantial performance improvements achieved through data augmentation. The trained models are subsequently validated through real-world field testing, confirming its accuracy and efficiency in practical environments.
4. Generative Model Generalization: The generative model’s ability to generalize was evaluated using datasets from various geographic regions, particularly examining its performance on Indian road datasets, and further tested on medical imaging data to assess its adaptability beyond road distress detection, demonstrating its robustness across diverse applications.
5. Deployment on Server-Class Hardware: The optimized model was successfully deployed on servers with varying performance capabilities after applying additional optimizations. This deployment demonstrated the model’s lightweight design, with substantial improvements in generation speed across different server configurations and suitability for road maintenance and monitoring systems.
6. Development of a Cloud-Based Database and Web-Based Demo: Generated images and detection results are stored and periodically organized for centralized management through cloud-based infrastructure. This database enables engineers and researchers to access annotated road distress data centrally and supports continuous improvement of the generative model by integrating feedback from real-world detection results, fostering an adaptive and self-improving system.

### RoadDiffBox framework design and image generation

**Fig. 2.**
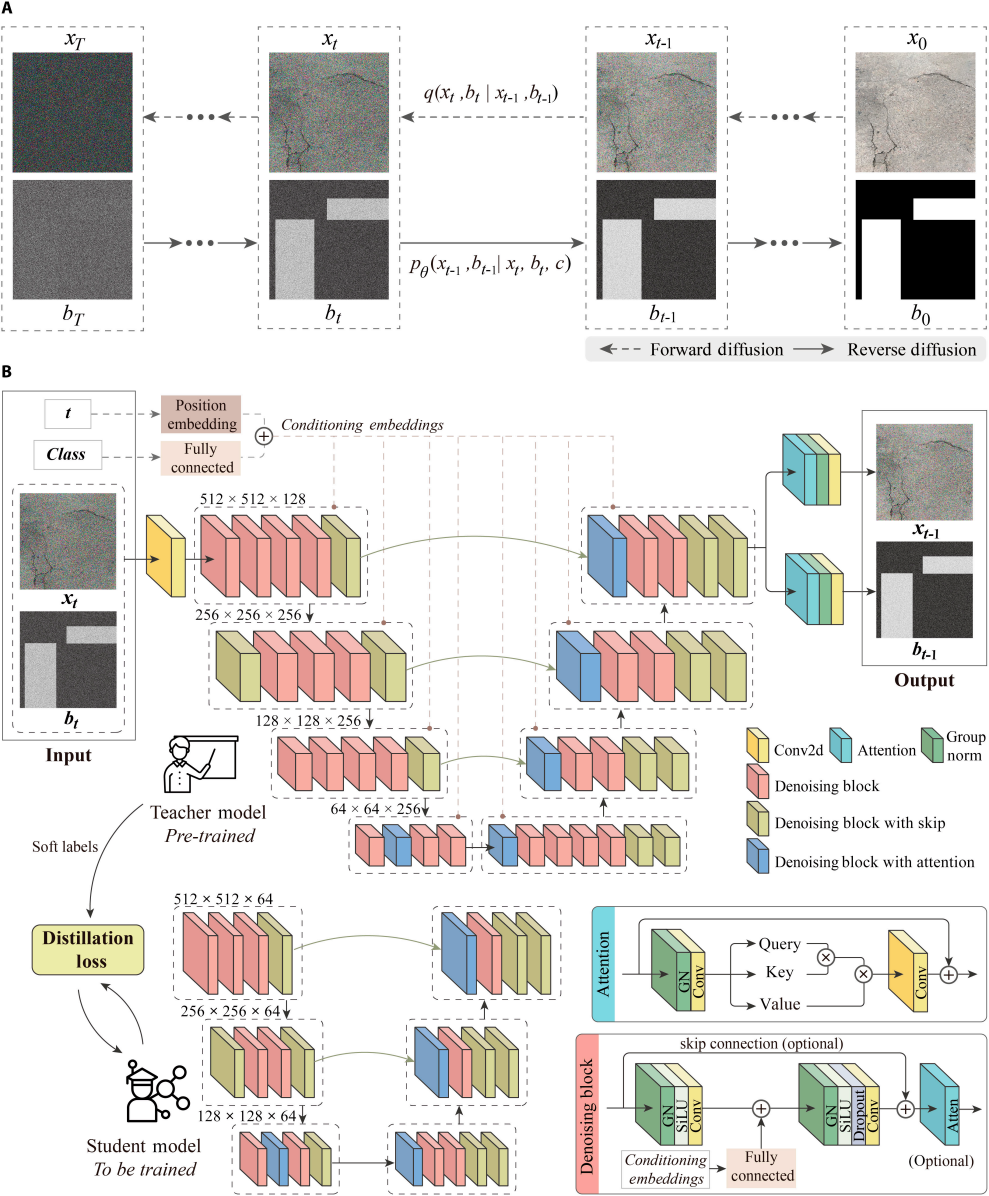
RoadDiffBox architecture: Diffusion processes and knowledge distillation. (A) Forward and reverse noise processes in diffusion model. (B) Architecture of teacher and lightweight student models.

As shown in Fig. 2, the RoadDiffBox framework addresses the specific challenges of road distress detection with emphasis on real-time performance and rapid deployment. This lightweight model enables precise class control across 4 distress categories (longitudinal cracks, alligator cracks, repaired pavement, and transverse cracks) while simultaneously generating corresponding bounding boxes. The architecture incorporates advanced techniques to enhance both generation efficiency and output quality. The automatically generated bounding boxes precisely localize distress areas and serve as ground truth for training detection models without manual annotation, effectively eliminating a major bottleneck in developing accurate detection systems. This approach meets the practical needs of transportation engineers by delivering engineering-level precision while maintaining a substantially reduced parameter count.

Figure 2A illustrates the core diffusion process in RoadDiffBox. During the forward diffusion phase, noise is gradually added to the input data, transforming clear images into random noise. In the reverse diffusion phase, the model progressively denoises the data, reconstructing realistic images from the noisy input [39]. To improve the efficiency of this reverse process, RoadDiffBox employs the DDIM method [40], which substantially accelerates sampling speed compared to traditional diffusion models, reducing the time required to generate images. Additionally, predefined labels are integrated into the reverse process, enabling the model not only to produce specific types of road distress images but also to generate corresponding bounding boxes. This ensures that the output images are realistic and annotated for direct use in detection tasks.

Figure 2B depicts the knowledge distillation process used for model compression within RoadDiffBox. Knowledge distillation plays a crucial role in reducing computational demands while maintaining model effectiveness, enabling efficient deployment in diverse environments [41]. By utilizing a teacher–student architecture, the large teacher model (60,489,476 parameters) transfers essential knowledge through soft labels, which represent the probability distribution over classes rather than discrete hard labels. Soft labels convey class relationships, allowing the student model to capture subtle distinctions and inter-class similarities, which improves generalization, mitigates overfitting, and enhances robustness to noisy data. As a result, the student model learns critical representations while substantially reducing complexity. The compressed student model, with only 5,040,799 parameters—a 91.67% reduction—preserves key functions such as class control and bounding box generation, ensuring high performance with lower computational resources. This approach enhances inference efficiency and reduces memory consumption while enabling real-time road distress detection on resource-constrained devices, supporting deployment across edge platforms, mobile applications, and cloud-based monitoring systems for scalable and intelligent road maintenance.

To further enhance the model’s generalization capability, self-attention mechanisms were incorporated into the architecture, as shown in Fig. 2B. These mechanisms enable the model to capture long-range dependencies within the input data, improving its ability to handle complex road distress patterns. This feature ensures robust performance across various geographic regions and road conditions, making RoadDiffBox adaptable to a range of real-world applications.

**Fig. 3.**
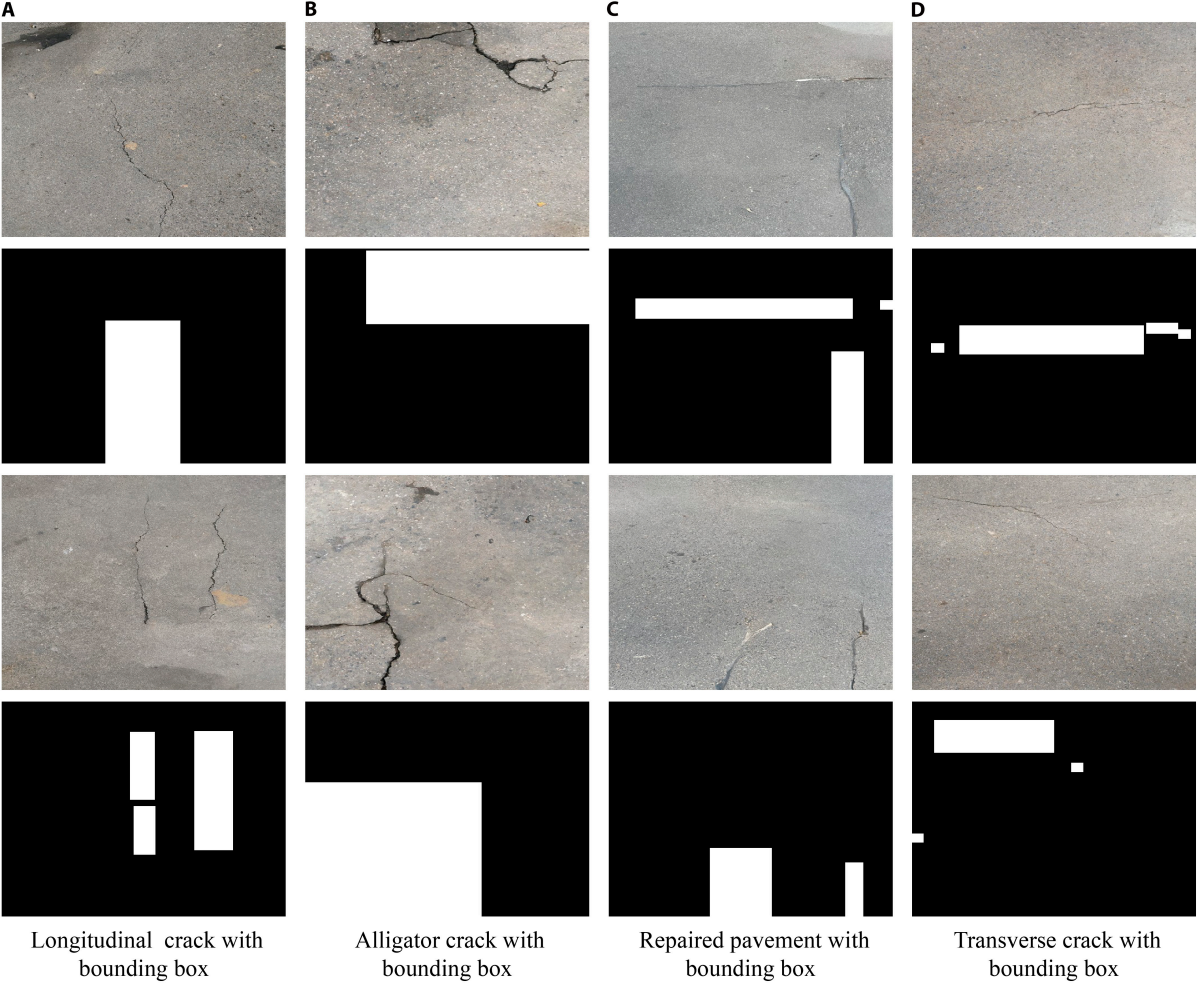
Category-controlled road distress images with generated bounding boxes. (A) Longitudinal crack, (B) alligator crack, (C) repaired pavement, and (D) transverse crack.

Following the establishment of the RoadDiffBox framework, its ability to generate class-controllable road distress images with corresponding labels was tested. Figure 3 shows the results, displaying 4 different categories of road distress images generated by RoadDiffBox, each with automatically generated bounding boxes. The black and white bounding boxes in the second and fourth rows indicate object localization, where white boxes specifically highlight the detected distress regions within the bounding area. This demonstrates the model’s precise control over crack-type generation, offering a versatile solution for creating diverse, annotated datasets suitable for various conditions.

### RoadDiffBox model performance evaluation

The performance of RoadDiffBox was evaluated through classification and object detection tasks to assess the quality and effectiveness of the generated synthetic data for training detection models. These evaluation methods have substantial physical significance, as they directly assess the model’s ability to identify distress types and precisely locate damaged areas on actual road surfaces—capabilities essential for real-world infrastructure maintenance decision-making. A key advantage of the proposed model is its ability to generate images paired with specific class labels and corresponding bounding boxes, enabling training without manual labeling. This approach facilitates semi-supervised learning, as the generated image–label pairs can automatically train detection models, by substantially lowering the need for manually labeled datasets.

**Fig. 4.**
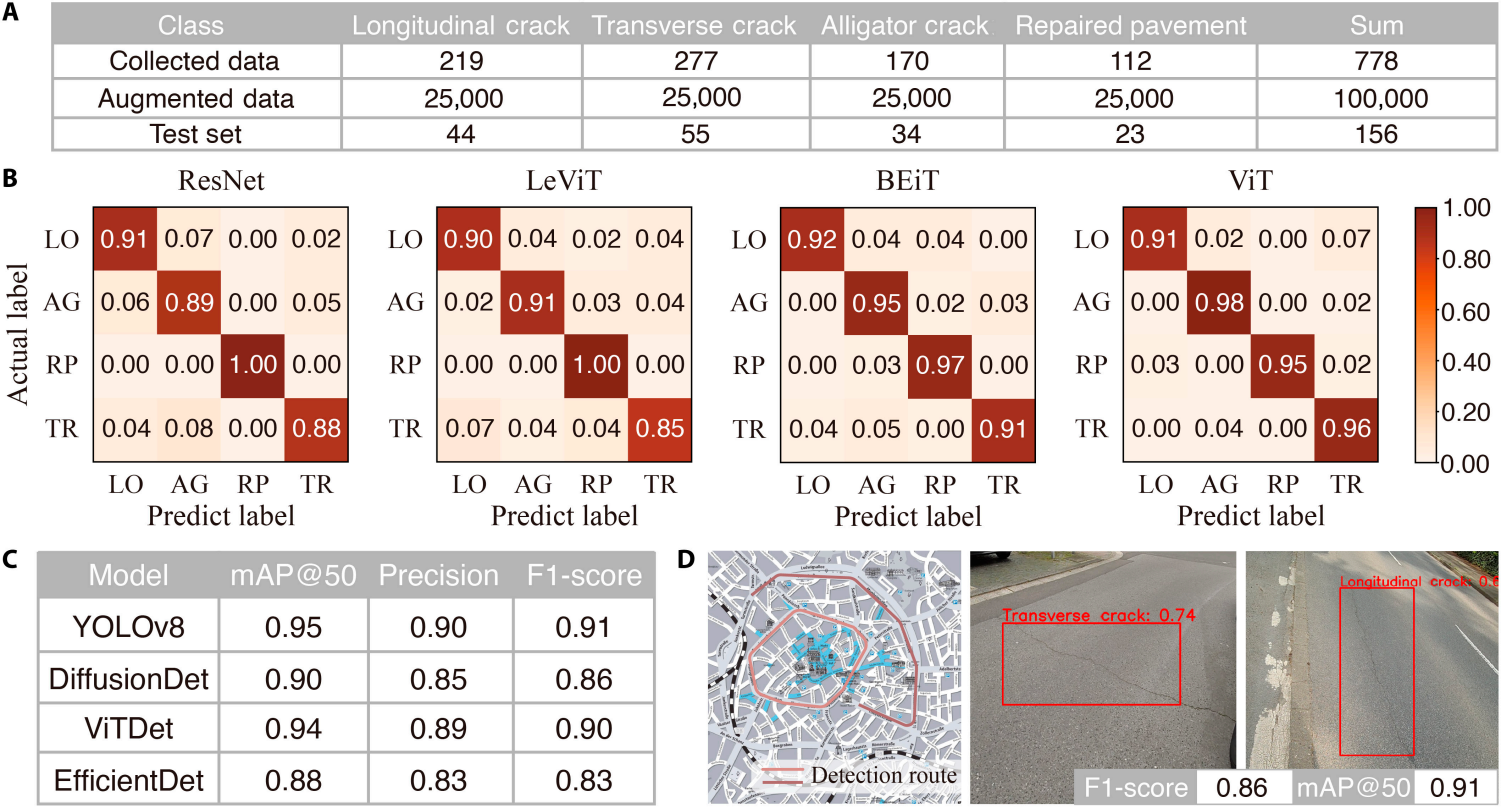
Performance evaluation of RoadDiffBox: Classification and object detection results. (A) Dataset used in experiments. (B) Confusion matrices for classification using ResNet, LeViT, BEiT, and ViT. LO, longitudinal cracks; AG, alligator cracks;p RP, repaired pavement; TR, transverse cracks. (C) Object detection performance of YOLOv8, DiffusionDet, ViTDet, and EfficientDet on generated bounding boxes. (D) On-site detection results using YOLOv8 in Aachen: Real-time testing. Map tiles adapted from Stamen Design under CC BY 3.0. Data © OpenStreetMap contributors (ODbL).

To demonstrate RoadDiffBox’s capabilities, the model generated 100,000 image–label pairs across 4 different road distress categories, with detailed dataset distribution shown in Fig. 4A. The effectiveness of these pairs was first assessed in classification tasks using 4 different models: ResNet [42], LeViT [43], BEiT [44], and ViT [45]. The confusion matrices presented in Fig. 4B highlight each model’s performance, with F1-scores as follows: ResNet (0.92), LeViT (0.91), BEiT (0.94), and ViT (0.95). These results confirm that RoadDiffBox can generate well-labeled data that are accurately classified across all 4 categories.

The effectiveness of the generated bounding boxes was further evaluated through object detection tasks. The generated dataset was used to train 4 advanced detection models: YOLOv8 [46], DiffusionDet [47], ViTDet [48], and EfficientDet [49], as shown in Fig. 4C. Each model’s performance was assessed using metrics such as mean average precision (mAP@50), precision, and F1-score. The detection task followed a 4-class classification scheme, distinguishing between longitudinal cracks, alligator cracks, repaired pavement, and transverse cracks, ensuring the evaluation captured class-specific performance. YOLOv8 performed best overall, achieving an mAP@50 of 0.95 and an F1-score of 0.91, which validates the accuracy and efficiency of the bounding boxes generated by RoadDiffBox for training object detection models.

To validate the detection model’s effectiveness under real-world conditions, an on-site detection test was conducted using the top-performing YOLOv8 model. This test took place in Aachen, Germany, with vehicles capturing images in real time as they traversed urban areas, covering a total of 568 images and 738 cracks. As shown in Fig. 4D, the model’s detection results were compared with manual inspection, achieving an F1-score of 0.86 and an mAP@50 of 0.91. These results demonstrate the robustness and applicability of RoadDiffBox in practical road distress detection tasks.

### Evaluation on datasets from different regions and cross-domain applications

The generalization capability of RoadDiffBox was evaluated on road distress datasets from different regions and tested on a medical skin lesion dataset, providing an assessment of its adaptability across specific domains.

**Fig. 5.**
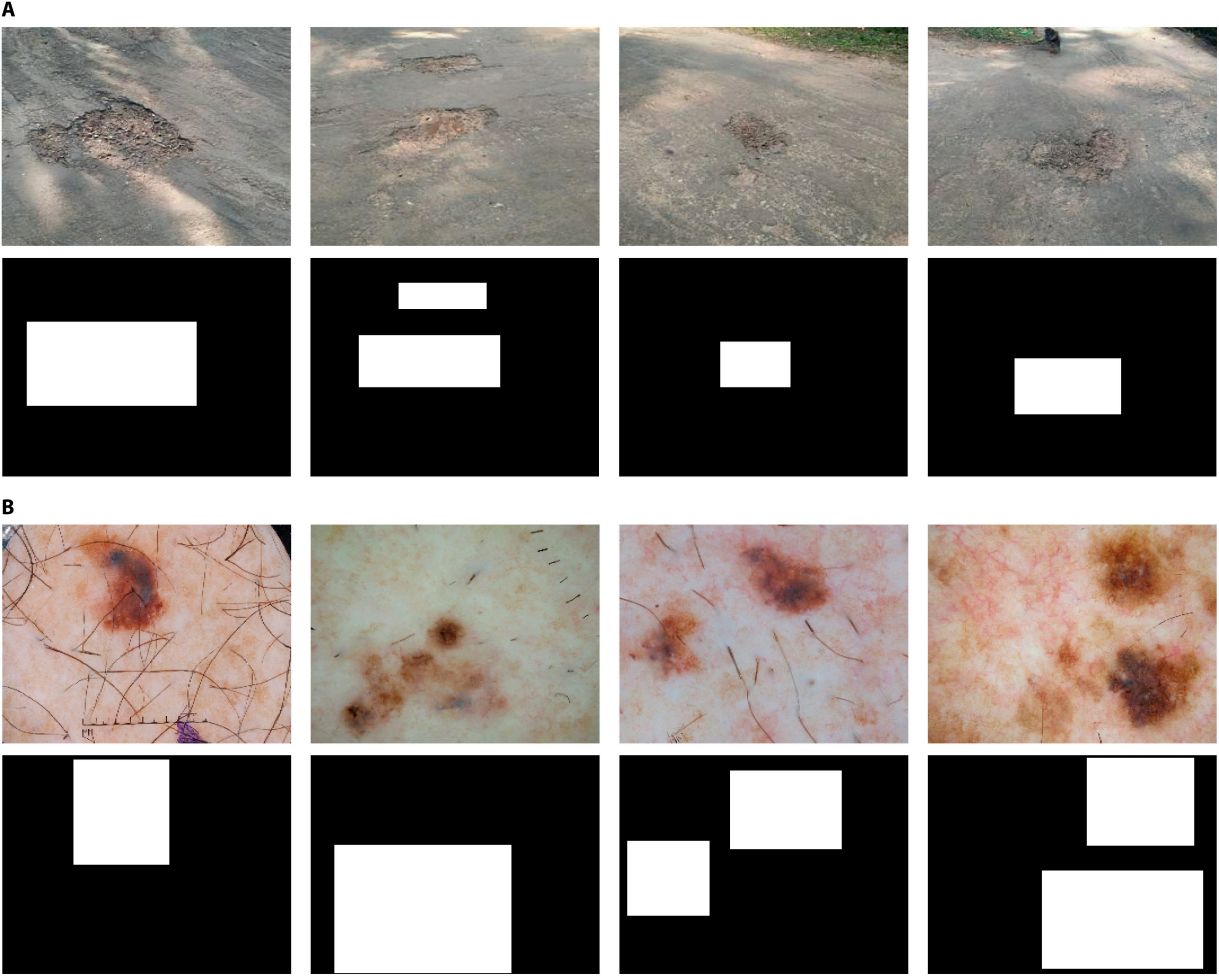
Generalization evaluation of RoadDiffBox: Same-domain and cross-domain results. (A) Generated road distress images and bounding boxes on the Indian pothole dataset. (B) Generated medical images and bounding boxes on the skin lesion dataset.

To assess the model’s performance within the same domain but across different geographic regions, RoadDiffBox was applied to an Indian road distress dataset consisting primarily of pothole images [50]. As shown in Fig. 5A, the model successfully generated realistic road distress images with accurate bounding box annotations. These results confirm that the model can adapt to geographic variations in road conditions, maintaining the ability to produce accurately annotated images despite regional differences in distress features.

For cross-domain testing, RoadDiffBox was applied to a medical dataset focused on skin lesion images [51]. As shown in Fig. 5B, the model was able to generate high-quality medical images with precise bounding box annotations. This demonstrates the model’s potential to extend beyond its original application in road distress detection and adapt to entirely different fields, such as medical imaging. While these cross-domain results are visually promising, it should be noted that further quantitative evaluation metrics would be helpful to precisely measure the generalization performance in these alternative domains.

In summary, RoadDiffBox demonstrated generalization capability when tested on road distress datasets from different regions and when applied to the medical skin lesion dataset. The incorporation of self-attention mechanisms in the model architecture contributed to its performance across these specific test conditions.

### Deployment of RoadDiffBox on server-class hardware

To further evaluate the practicality and efficiency of RoadDiffBox across different levels of computational power, the model was deployed on various server-grade GPUs after optimization through FP16 quantization and TensorRT conversion. These optimization techniques were implemented to reduce the model’s memory footprint and computational requirements while maintaining detection accuracy. FP16 quantization reduces the precision of floating-point calculations from 32 to 16 bits, effectively halving memory usage and increasing processing speed [52], while TensorRT conversion optimizes the model for NVIDIA GPUs by fusing operations and selecting efficient kernel implementations [46]. These optimizations were essential to ensure efficient operation on a range of hardware configurations with varying resources, making RoadDiffBox deployable in both resource-constrained field devices and high-performance server environments.

The optimized RoadDiffBox model was tested on 4 different GPUs: RTX 4090, RTX 3080 Ti, RTX 2080 Ti, and RTX 3060, which were selected to represent a range of high-end, mid-range, and previous-generation GPUs commonly used in real-world engineering applications. The RTX 4090 serves as a high-performance reference, the RTX 3080 Ti and RTX 2080 Ti reflect widely used research and industrial setups, while the RTX 3060 represents resource-constrained deployment scenarios. The deployment aimed to assess generation speeds across devices with different performance capabilities. For benchmarking, the time required to generate an image and its corresponding bounding box annotation was measured. Additionally, 2 different batch sizes (1 and 2) were tested to maximize utilization of the computing resources available on each device, and the time required per image was recorded.

**Fig. 6.**
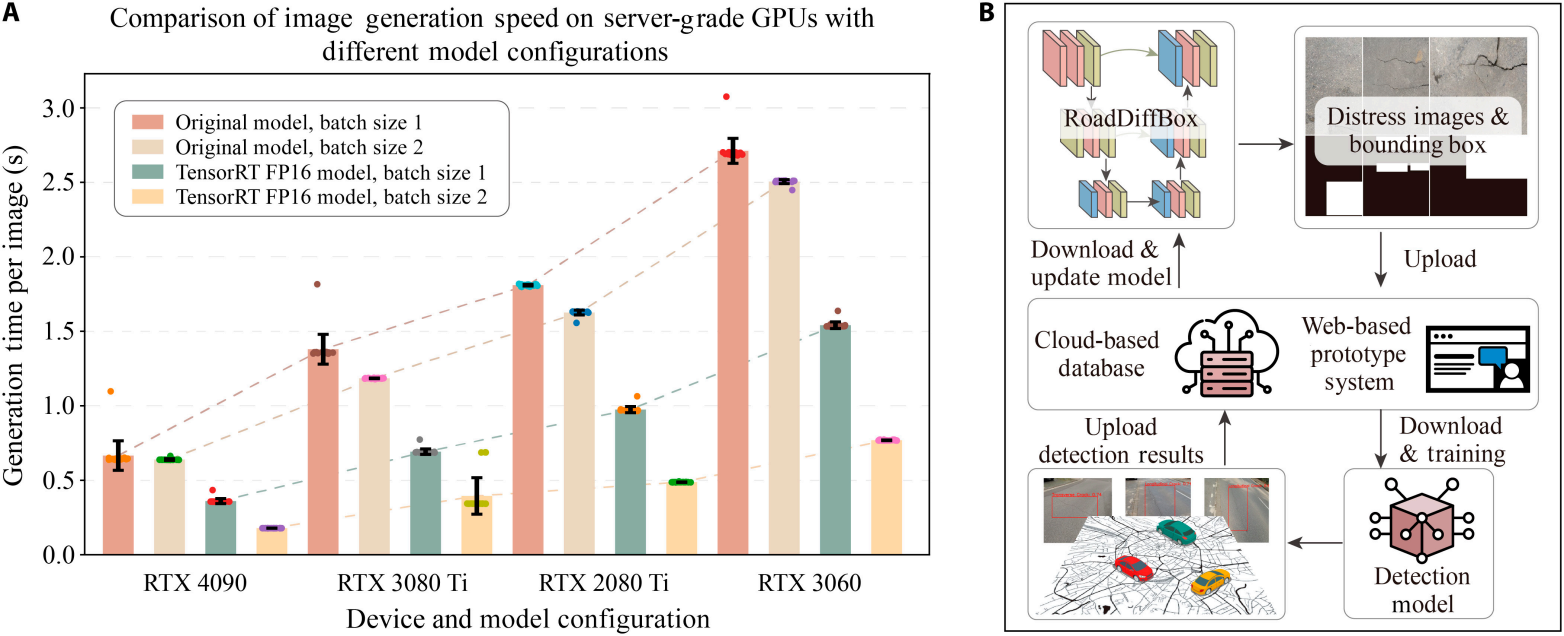
RoadDiffBox performance on server-grade GPUs and cloud-based integration. (A) Comparison of image generation speed on server-grade GPUs with different model configurations. (B) Cloud-based database for centralized data storage and model improvement. Icons by shashank singh and Matt Saling from The Noun Project (CC BY 3.0). Map tiles adapted from stamen design under CC BY 3.0. Data © OpenStreetMap contributors (ODbL).

The results, as shown in Fig. 6A, highlight the model’s efficiency across various GPUs. The RTX 4090, the most powerful device tested, achieved the fastest generation time per image–label pair, averaging just 0.18 s. The RTX 3060, although less powerful and more cost-effective, generated an image in 0.77 s—a performance still suitable for real-world engineering applications that require efficient image generation without extensive hardware investment. These results demonstrate that the lightweight optimizations applied to RoadDiffBox make it highly versatile, capable of rapid image generation even on less powerful hardware.

### Development of a cloud-based database and web-based prototype system for RoadDiffBox

In addition to deploying the RoadDiffBox model for road distress detection, a cloud-based database was constructed to systematically store and manage the model’s generated and detected data. This database is designed to enable centralized data management and provide standardized access to annotated road distress datasets for research and practical applications.

As illustrated in Fig. 6B, the outputs generated by RoadDiffBox, including synthesized road distress images and bounding box annotations, are organized within this database for centralized storage and systematic management. This approach will enable centralized access to high-quality, annotated road distress data, benefiting a broad audience of practitioners, researchers, and policymakers engaged in infrastructure management. Additionally, the database incorporates detection results from field-deployed models as part of a self-improving feedback system. These detection outputs are periodically fed back into the training pipeline of the diffusion model, allowing it to adapt and improve its generative capabilities over time. This iterative cycle not only enhances the quality of the synthetic data generated but also progressively refines the accuracy and robustness of the detection models, fostering a sustainable feedback loop for continuous model improvement.

Additionally, a web-based prototype system of RoadDiffBox was developed, providing road maintenance professionals worldwide with a hands-on experience of the model’s capabilities. This platform allows users to explore real-time road distress image generation through an intuitive interface, where they can specify the type of road distress and view the resulting images, complete with bounding box annotations. Access the prototype system here: http://skingserver.top:44455. This interactive tool offers users a practical glimpse into the potential of RoadDiffBox to enhance road maintenance workflows.

## Discussion

This study introduces a lightweight diffusion model, RoadDiffBox, specifically designed for generating road distress images. By utilizing DDIM sampling, the model achieves faster generation speeds while incorporating architecture modifications to enable class control, producing images with corresponding bounding boxes. Additionally, the integration of self-attention mechanisms enhances RoadDiffBox’s generalization ability, allowing it to adapt to diverse road conditions across regions. The flexibility of this framework—especially its ability to generate annotated datasets without manual intervention—makes it an ideal tool for implementing real-time road monitoring and maintenance in data-limited and computational resources-limited scenarios.

Performance evaluations have confirmed the effectiveness of RoadDiffBox in generating high-quality image–label pairs. By employing semi-supervised learning, the model substantially reduces reliance on labeled data. The trained model maintains high accuracy in classification and object detection tasks, with on-site experiments further validating the detection model’s effectiveness. Results demonstrate that RoadDiffBox can generate well-labeled data across multiple road distress categories, providing precise bounding boxes for detection models.

Generalization testing showed RoadDiffBox’s ability to process road distress data from different geographic contexts, including successful application to the Indian pothole dataset. The examination of cross-domain application to skin lesion images suggests potential utility beyond road infrastructure, though further domain-specific validation would be required for conclusive assessment of other specialized applications.

For deployment, RoadDiffBox was optimized and successfully implemented on various server-class hardware using TensorRT conversion and FP16 quantization. Results showed a substantial reduction in generation time, with the fastest server (RTX 4090) producing each image–label pair in just 0.18 s, while the more cost-effective RTX 3060 achieved a generation time of 0.77 s per image. These improvements in speed and efficiency demonstrate RoadDiffBox’s adaptability for real-time, cost-effective applications across a range of server configurations, underscoring its practicality for road monitoring systems that require rapid, accurate image generation.

Finally, a cloud-based database was developed to support continuous improvement of the RoadDiffBox framework. The database systematically stores the generated images and detection results, providing centralized access to annotated road distress data through cloud-based infrastructure. Additionally, a web-based demo was created to offer road maintenance professionals an interactive experience, enabling them to specify distress types and view generated images with bounding box annotations in real time. This comprehensive infrastructure delivers 2 key advantages. A feedback loop uses field detection results to continuously enhance the generative model. Additionally, maintenance teams gain broader access to advanced distress detection technology. Together, these benefits enable more responsive and cost-effective infrastructure management strategies.

RoadDiffBox addresses the fundamental challenge of data scarcity and high acquisition costs that have long hindered the development of intelligent road infrastructure monitoring systems. The framework substantially enhances detection model accuracy through data augmentation, enabling more precise automated road assessment and evaluation processes. By generating comprehensive synthetic datasets with accurate annotations, RoadDiffBox facilitates the integration of advanced detection capabilities into pavement management systems, potentially improving the accuracy of pavement condition index assessments. The enhanced data availability and detection precision contribute to more accurate decision-making processes in road maintenance scheduling and resource allocation. The AIGC method demonstrated here holds broader importance, with potential applications in fields such as construction, satellite imaging, medical imaging, and aerospace, where large-scale annotated datasets are essential for monitoring and detection tasks.

## Methods

### Data collection

This study utilized 3 distinct datasets to comprehensively evaluate RoadDiffBox’s performance, cross-regional generalization, and cross-domain applicability.

The first dataset is used for training the RoadDiffBox model and aims to capture various road distress conditions from different geographic regions. To enhance dataset robustness, images were collected from Germany (Aachen) and China (Beijing), where road distress characteristics differ due to varied environmental and traffic conditions. Aachen experiences moderate seasonal temperature variations and high traffic intensity, particularly on arterial roads and highways, leading to distress patterns influenced by both thermal fluctuations and heavy dynamic loads. Beijing, characterized by extreme seasonal temperature shifts, undergoes pronounced thermal expansion and contraction cycles, impacting pavement durability, especially in resurfaced layers. This dataset includes 778 images covering 4 distinct categories, each annotated with bounding boxes indicating crack locations. While comprehensive, the dataset size remains relatively limited, and additional geographic diversity, as well as variations in imaging conditions such as weather, lighting, and road materials, could further enhance model generalization and adaptability to diverse real-world scenarios.

The second dataset evaluates the model’s generalization capability for cross-regional road distress detection. For this purpose, a pothole image dataset containing 245 images from India was used [50]. To align with the model’s output format, segmentation masks in this dataset were converted to bounding box annotations. This dataset is crucial for testing the model’s adaptability across different geographic regions.

The third dataset assesses the model’s cross-domain generalization ability. For this, the International Skin Imaging Collaboration Challenge dataset [51,53], consisting of 2,594 skin lesion images from the medical field, was used. Similar to the second dataset, segmentation information was converted to bounding box annotations to meet the model’s training requirements. This dataset enables an evaluation of RoadDiffBox’s versatility, demonstrating its ability to generate accurately labeled images in an entirely different domain, highlighting its potential beyond road distress detection.

### Experiment environment

The RoadDiffBox model was trained on a system running Ubuntu 22.04, equipped with 4 RTX 4090 GPUs, each with 48 GB of VRAM, using Python 3.8 and PyTorch 2.1. The server GPUs used for deployment included the RTX 4090 (16,384 CUDA cores, 24 GB GDDR6X, 82.6 TFLOPS), RTX 3080 Ti (10,240 CUDA cores, 12 GB GDDR6X, 34.1 TFLOPS), RTX 2080 Ti (4,352 CUDA cores, 11 GB GDDR6, 13.4 TFLOPS), and RTX 3060 (3,584 CUDA cores, 12 GB GDDR6, 12.7 TFLOPS).

### Diffusion model

The RoadDiffBox model is based on the diffusion process [39], which includes 2 key phases: forward diffusion and reverse diffusion. Through this diffusion process, the model can generate road distress images with controllable categories and corresponding bounding box annotations.

In the forward diffusion phase, noise is gradually added over several time steps to both the road distress image and its bounding box, transforming the input into a noisy version. At each time step t, the model generates noisy versions of the image $x_{t}$ and bounding box $b_{t}$. The forward diffusion process is defined as follows [39]:

$$\begin{array}{c} q\left(x_{t},\;b_{t}|\;x_{t-1},\;b_{t-1}\right)=\\ N\left(x_{t};\;\sqrt{1-\beta_{t}}x_{t-1},\;\beta_{t}I\right)N\left(b_{t};\;\sqrt{1-\beta_{t}}b_{t-1},\;\beta_{t}I\right) \end{array}$$ (1)

where $\beta_{t}$ is a variance schedule that controls the amount of noise added at each step, and I is the identity matrix, ensuring that noise is applied isotropically (uniformly in all directions). This process gradually adds Gaussian noise to both the image and bounding box annotations, until they converge to pure noise at the final time step T.

The reverse diffusion phase aims to recover the original image and bounding box by progressively removing the noise introduced in the forward phase. The model learns the reverse conditional distribution [39]:

$$\begin{array}{c} \begin{array}{c} \begin{array}{c} p_{\theta }\left(x_{t-1},\;b_{t-1}|\;x_{t},\;b_{t},\;c\right)=\\ N\left(x_{t-1};\;\mu_{\theta }\left(x_{t},\;t,\;c\right),\;\Sigma_{\theta }\left(x_{t},\;t,\;c\right)\right)N\left(b_{t-1};\;\mu_{\theta }\left(b_{t},\;t,\;c\right),\;\Sigma_{\theta }\left(b_{t},\;t,\;c\right)\right) \end{array} \end{array} \end{array}$$ (2)

where c is the class control input, which allows the model to generate specific types of road distress images, such as longitudinal or reflective cracks. In this equation, $\mu_{\theta }$ and $\Sigma_{\theta }$ represent the model’s predicted mean and variance for the image and bounding box at each step, conditioned on class c.

To further accelerate the reverse diffusion process, the DDIM method was applied. This method reduces the number of reverse diffusion steps by providing a deterministic mapping between noisy images and their denoised counterparts, as well as their bounding boxes. For image $x_{t}$ and bounding box $b_{t}$, the DDIM reverse process is defined as [40]:

$$\begin{array}{c} x_{t-1}=\sqrt{\alpha_{t-1}}\left(\frac{x_{t}-\sqrt{1-\alpha_{t}}\epsilon_{\theta }\left(x_{t},\;t\right)}{\sqrt{\alpha_{t}}}\right)+\sqrt{1-\alpha_{t-1}}\epsilon_{\theta }\left(x_{t},\;t\right) \end{array}$$ (3)

$$\begin{array}{c} b_{t-1}=\sqrt{\alpha_{t-1}}\left(\frac{b_{t}-\sqrt{1-\alpha_{t}}\epsilon_{\theta }\left(b_{t},\;t\right)}{\sqrt{\alpha_{t}}}\right)+\sqrt{1-\alpha_{t-1}}\epsilon_{\theta }\left(b_{t},\;t\right) \end{array}$$ (4)

where $\alpha_{t}$ is the noise schedule, and $\epsilon_{\theta }$ represents the model’s predicted noise at each step for both the image and bounding box. This approach substantially speeds up the generation process while preserving the quality of both the image and bounding box.

The model was trained over 20 million iterations with a learning rate of 1×10^−4^ and a batch size of 16, ensuring efficient convergence and robust performance.

### Knowledge distillation

To construct the RoadDiffBox model, knowledge distillation [41] was applied. Knowledge distillation is a technique that transfers knowledge from a larger, more complex model (the teacher model) to a smaller, more efficient model (the student model). The main objective of this process is to enable the student model to achieve performance comparable to the teacher model while substantially reducing the number of parameters and computational requirements.

In the distillation process, the teacher model is first trained on the dataset, and the student model is then trained to mimic its output. Traditionally, models are trained using hard labels, where each sample is assigned a single discrete class, treating all incorrect classes as equally distant from the correct one [41]. While effective, this approach can lead to overfitting and poor generalization, especially in cases with ambiguous or noisy data. Instead, the student model in knowledge distillation learns from soft labels generated by the teacher model. Soft labels represent a probability distribution over all possible classes rather than a single categorical value, conveying relative confidence levels across different classes [41]. This provides richer information about class relationships and uncertainty, allowing the student model to capture finer distinctions, improve generalization, and enhance robustness to noise. By utilizing these more informative targets, the student model effectively retains essential knowledge while achieving substantial reductions in computational complexity.

In this work, mean squared error (MSE) loss [54] was used as the distillation loss to measure the difference between the predictions of the student and teacher models. The objective is to minimize the MSE between the teacher’s output $z_{\text{teacher}}$ and the student’s output $z_{\text{student}}$, expressed as:

$$\begin{array}{c} \text{MSE}\;\text{Loss}=\frac{1}{n}\sum_{i=1}^{n}{\left(z_{\text{teacher}}^{\left(i\right)}-z_{\text{student}}^{\left(i\right)}\right)}^{2} \end{array}$$ (5)

where n is the number of predictions, and $z_{\text{teacher}}^{\left(i\right)}$ and $z_{\text{student}}^{\left(i\right)}$ represent the outputs of the teacher and student models, respectively.

By applying this distillation loss, the student model effectively learns from the teacher model and approximates its performance while substantially reducing the number of parameters. In this implementation, the student model achieved a 91.67% reduction in parameters compared to the teacher model, while maintaining similar accuracy in image generation and bounding box prediction.

### Evaluation metrics

To comprehensively evaluate the performance of the RoadDiffBox model, several key metrics tailored for classification and detection tasks were used. These metrics help assess the model’s ability to generate accurately labeled road distress images and bounding box annotations.

For classification tasks, a confusion matrix [55] and F1-score [56] were used as evaluation metrics. For detection tasks, metrics included mAP@50 [57], precision, and F1-score.

The F1-score, a harmonic mean of precision and recall, provides a balanced measure of model performance, especially useful in cases of class imbalance. It is defined as:

$$F1-\text{score}=2\times \frac{\text{Precision}\times \text{Recall}}{\text{Precision}+\text{Recall}}$$ (6)

mAP@50 assesses the overlap between predicted and ground-truth bounding boxes, with an intersection over union (IoU) threshold of 0.50. The IoU formula is:

$$\mathrm{IoU}=\frac{\text{Area of overlap}}{\text{Area of union}}$$ (7)

mAP@50 is calculated by averaging the precision across different recall levels when IoU is at least 0.50.

Precision measures the proportion of true positive predictions among all positive predictions, making it a valuable metric for both classification and detection tasks. It is defined as:

$$\text{Precision}=\frac{\mathrm{TP}}{\mathrm{TP}+\mathrm{FP}}$$ (8)

Using these metrics, the RoadDiffBox model’s performance in both classification and detection was thoroughly evaluated, ensuring accurate predictions in image classification and bounding box generation tasks.

### Semi-supervised learning for road distress detection

Semi-supervised learning is a machine learning paradigm that leverages both labeled and unlabeled data to enhance model performance while reducing dependence on extensive manual annotations [58]. Traditional semi-supervised learning approaches, such as pseudo-labeling and consistency regularization, enable models to learn from a small set of labeled samples while extracting meaningful patterns from unlabeled data. This strategy is particularly useful in applications where large-scale annotation is costly and labor-intensive.

In this study, semi-supervised learning is defined as a training strategy that eliminates the need for additional manual annotation by directly utilizing the automatically generated dataset from RoadDiffBox. Unlike conventional semi-supervised learning methods that require human-annotated seed datasets, the proposed framework benefits from RoadDiffBox’s ability to generate synthetic road distress images along with corresponding bounding box annotations. The generated dataset can be seamlessly integrated into object detection model training, creating a semi-supervised learning pipeline that does not rely on additional labeling efforts.

The semi-supervised learning process begins with RRoadDiffBox generating a diverse dataset of road distress images, each automatically annotated with bounding boxes. These synthetic images complement real-world labeled data, expanding the distribution of distress patterns encountered during training. A base object detection model, pre-trained on real-world samples, is further refined using the generated dataset, enabling it to learn from both real and synthetic data. By leveraging RoadDiffBox’s annotations, the model improves its ability to generalize to unseen road conditions, while self-training further enhances feature learning and reduces dependence on manual labeling.

### RoadDiffBox model deployment on server-class hardware

To ensure that the RoadDiffBox model could be effectively deployed on server-class hardware for real-time road condition detection, a series of optimization steps were implemented. First, FP16 quantization was applied, reducing the model’s computational precision from FP32 to FP16 (16-bit floating point). This reduction substantially lowered memory requirements and computational load, which is essential for efficient operation on a variety of server configurations.

Next, the quantized model was converted to TensorRT format. This conversion introduced key performance improvements through techniques such as layer fusion, precision calibration, and kernel auto-tuning, which greatly reduced inference time. These optimizations allowed the model to operate efficiently across different NVIDIA GPUs.

The optimized model was then deployed on 4 server GPUs: RTX 4090, RTX 3080 Ti, RTX 2080 Ti, and RTX 3060, each with distinct performance capabilities. This deployment enabled a comprehensive evaluation of the model’s efficiency across various hardware configurations, from high-performance to more cost-effective, energy-efficient options. The results validated the model’s adaptability and scalability, demonstrating its suitability for road monitoring applications on servers with varying computational resources.

## Data Availability

The datasets and code supporting this study are openly available at http://skingserver.top:44455. Further details and inquiries can be directed to the corresponding author.
